# Seventeen Ustilaginaceae High-Quality Genome Sequences Allow Phylogenomic Analysis and Provide Insights into Secondary Metabolite Synthesis

**DOI:** 10.3390/jof8030269

**Published:** 2022-03-08

**Authors:** Lena Ullmann, Daniel Wibberg, Tobias Busche, Christian Rückert, Andreas Müsgens, Jörn Kalinowski, Lars M. Blank

**Affiliations:** 1 iAMB—Institute of Applied Microbiology, ABBt—Aachen Biology and Biotechnology, RWTH Aachen University, Worringerweg 1, 52074 Aachen, Germany; lena.ullmann@rwth-aachen.de (L.U.); andreas.muesgens@rwth-aachen.de (A.M.); 2 Center for Biotechnology (CeBiTec), Bielefeld University, Universitätsstraße 27, 33615 Bielefeld, Germany; dwibberg@cebitec.uni-bielefeld.de (D.W.); tbusche@cebitec.uni-bielefeld.de (T.B.); cruecker@cebitec.uni-bielefeld.de (C.R.); joern@cebitec.uni-bielefeld.de (J.K.)

**Keywords:** AAI, ANI, POCP, Oxford nanopore, phylogenomics, Ustilaginaceae, metabolic engineering, itaconic acid, ustilagic acid, smut fungi, *Ustilago maydis*

## Abstract

The family of Ustilaginaceae belongs to the order of Basidiomycetes. Despite their plant pathogenicity causing, e.g., corn smut disease, they are also known as natural producers of value-added chemicals such as extracellular glycolipids, organic acids, and polyols. Here, we present 17 high-quality draft genome sequences (N50 > 1 Mb) combining third-generation nanopore and second-generation Illumina sequencing. The data were analyzed with taxonomical genome-based bioinformatics methods such as Percentage of Conserved Proteins (POCP), Average Nucleotide Identity (ANI), and Average Amino Acid Identity (AAI) analyses indicating that a reclassification of the Ustilaginaceae family might be required. Further, conserved core genes were determined to calculate a phylogenomic core genome tree of the Ustilaginaceae that also supported the results of the other phylogenomic analysis. In addition, to genomic comparisons, secondary metabolite clusters (e.g., itaconic acid, mannosylerythritol lipids, and ustilagic acid) of biotechnological interest were analyzed, whereas the sheer number of clusters did not differ much between species.

## 1. Introduction

The family of Ustilaginaceae belongs to the order of Ustilaginomycetes. It consists of 14 genera, including *Ustilago, Sporisorium*, and *Macalpinomyces* [1]. The taxonomy of the Ustilaginaceae, which is still a matter of debate, was focused on during several studies resulting in several taxonomic revisions [2]. For instance, Kirk et al. (2008) proposed that the Ustilaginaceae family comprises 17 genera comprising 607 species [3]. The ever-increasing genomic data should settle the discussion at one point. Ustilaginaceae are known for their plant pathogenicity and the capability to infect economically essential crops, including barley, sugarcane, wheat, and oats [3]. Even though these plant diseases cause crop loss, smut fungi attracted particular attention in the field of industrial biotechnology.

Ustilaginaceae show a versatile product spectrum including organic acids (e.g., itaconate, malate, succinate), polyols (e.g., erythritol, mannitol), and extracellular glycolipids, which are considered value-added chemicals with potential applications in the pharmaceutical, food, and chemical industries [4,5]. For instance, mannosylerythritol lipids (MELs) are surface-active glycolipids secreted by various fungi, including *U. maydis*. MELs can be used as biosurfactants, displaying the much-asked biodegradability resource for the production of detergents or pharmaceuticals [6]. The various organic acids produced by Ustilaginaceae are known as platform chemicals, which have the potential to contribute to the transition from a fossil- to a bio-based economy [7]. 

*U. maydis* does not only show a broad product spectrum, but also metabolizes a range of renewable carbon sources, which, apart from sugars, also include the monomers acetate, galacturonic acid, glycerol, and xylose, as well as the polymers cellulose, pectin, lignin, and xylan [8,9,10,11]. Among the Ustilaginaceae, *U. maydis* is the best-studied species in plant pathogenicity and biotechnology, and is a model organism in cell biology [12]. A broad range of molecular, genetic, bioinformatics, and cell biological techniques have been developed over the past years [13,14,15]. This, along with their yeast-like growth, makes Ustilaginaceae attractive for biotechnological applications.

Recent studies have advanced our understanding of the smut fungi by elucidating complete genome sequences of *U. maydis* and other members of the Ustilaginaceae family [16]. With a particular interest in itaconate producers, Geiser et al. (2016) compared nine different strains and observed that the synteny of the itaconate cluster is preserved in the investigated Ustilaginaceae [17]. Furthermore, the sequencing revealed genome sizes between 19 and 25 Mb of the investigated Ustilaginaceae via Illumina short-read sequencing.

Technological progress and the development of third-generation sequencing methods, e.g., nanopore sequencing, allow for the rapid and cost-efficient high-throughput generation of complete genome sequences with long reads [18]. Thus, next-generation sequencing displays a valuable tool for the investigation of phylogenomic and population genomic relationships, e.g., as shown previously in the investigation of Hypoxylaceae by Wibberg et al. (2021) [19]. 

In this study, 17 strains of the Ustilaginaceae were genomically analyzed by establishing high-quality draft genomes (N50 > 1 Mb) combining Oxford Nanopore and Illumina sequencing. Various comparative genomics methods, including Average Amino Acid Identity (AAI), Average Nucleotide Identity (ANI), and Percentage of Conserved Proteins (POCP), were used to estimate the similarity of the selected isolates and to compute a phylogenomic core genome tree of the Ustilaginaceae. In more detailed analyses, the presence of secondary metabolite gene clusters was investigated for potential biotechnological exploitation. With these high-quality draft genomes, a data set was established, enabling the investigation of Ustilaginaceae biodiversity, as well as contributing to biotechnological applications, e.g., metabolic engineering of secondary metabolite production.

## 2. Materials and Methods

### 2.1. Selection of Fungal Strains

For genome sequencing, 17 strains of the Ustilaginaceae family were chosen (Supplementary Table S1). Eleven representatives of the *Ustilago* genus (*U. curta*, *U. cynodontis*, *U. lituana*, *four U. maydis strains*, *U. trichophora*, *U. vetiveriae*, and *U. xerochloae*), and species of three other genera *(Sporisorium*, *Pseudozyma*, *Macalpinomyces*) were chosen. *Ustanciosporium gigantosporium* of the order of Ustilaginales represents the taxonomic outgroup.

### 2.2. Genomic DNA Preparation

Cells from frozen glycerol stocks were grown on YEP agar plates at 30 °C for 24–48 h. Single colonies of the fungal strains were grown in modified Tabuchi medium according to Geiser et al. [20] at 30 °C for 24 h (300 rpm, 80% humidity, d = 50 mm, Infors HT Multitron Pro shaker, Bottmingen, Switzerland). Cells were disrupted using type C bead tubes (Macherey-Nagel, Düren, Germany) for 25 min at full speed horizontally on a flat-bed vortexer (Vortex-Genie 2, Scientific Industries, New York, NY, USA). Genomic DNA was isolated by NucleoBond High molecular weight DNA Kit (Macherey-Nagel, Düren, Germany) according to the manufacturer’s instructions. 

### 2.3. Nanopore Library Preparation and GridION^®^ Sequencing

A sequencing library with genomic DNA from the different fungal strains was prepared using the Nanopore Rapid DNA Sequencing kit (SQK-RAD04, Oxford Nanopore Technologies, Oxford, UK) according to the manufacturer’s instructions. Sequencing was performed on an Oxford Nanopore GridION Mk1 sequencer using an R9.4.1 flow cell, which was prepared according to the manufacturer’s instructions.

### 2.4. Illumina Library Preparation and MiSeq Sequencing

Whole-genome-shotgun PCR-free libraries were constructed from 5 μg of gDNA with the Nextera XT DNA Sample Preparation Kit (Illumina, San Diego, CA, USA) according to the manufacturer’s protocol. Quality of the resulting libraries was controlled by using an Agilent 2000 Bioanalyzer with an Agilent High Sensitivity DNA Kit (Agilent Technologies, Santa Clara, CA, USA) for fragment sizes of 500–1000 bp. Paired-end sequencing was performed on the Illumina MiSeq platform (2 × 300 bp, v3 chemistry). Adapters and low-quality reads were removed by an in-house software pipeline prior to polishing as recently described [21].

### 2.5. Base Calling, Reads Processing, and Assembly

MinKNOW (Oxford Nanopore Technologies) was used to control the run using the 48 h sequencing run protocol; base calling was performed offline using Bonito. The assembly was performed using canu v2.1.1 [22]. The resulting contigs were polished with Illumina short-read data using Pilon [23] run for ten iterative cycles. BWA-MEM [24] was used for read mapping in the first five iterations and Bowtie2 v2.3.2 [25] in the second set of five iterations.

### 2.6. Gene Prediction and Genome Annotation

Gene prediction was performed by applying GeneMark-ES 4.6.2 [26] using default settings. Predicted genes were functionally annotated using a modified version of the genome annotation platform GenDB 2.0 [27] for eukaryotic genomes as previously described [28]. For automatic annotation within the platform, similarity searches against different databases, including COG [29], KEGG [30], and SWISS-PROT [31], were performed.

In addition to genes, putative tRNA genes were identified with tRNAscan-SE [32]. Completeness, contamination, and strain heterogeneity were estimated with BUSCO (v3.0.2 [33]), using the fungi-specific single-copy marker genes database (odb9). The obtained genome sequences were deposited in the DDBJ/EMBL/GenBank database under the accession numbers summarized in Table 1.

### 2.7. Comparative Genome Analyses and Phylogenetic Analysis

The genomes of the sequenced and annotated fungal strains were used for comparative genome analyses. Comparative analyses between fungal genomes were accomplished using a modified version of the comparative genomics program EDGAR designed to handle eukaryotic genomes and their multi-exon genes [34,35] as described recently [36]. Within the EDGAR workflow, classification of genes as core genes (genes shared in all genomes within a tested set) or singletons was performed based on BLAST Score Ratio Values (SRVs) and visualized in a Venn diagram. In addition, Average Nucleotide Identity (ANI) and Average Amino Acid Identity analyses (AAI) were performed based on the GeneMark prediction similarly to previously described methods [36] to determine the relationship between the different species. Multiple alignments for all core protein sequences were created using MUSCLE [37]. Pairwise Percentage of Conserved Proteins (POCP) analysis was performed according to [38] and as previously described [19,39,40].

For the AAI method, the percent identity values between amino acid sequences of orthologous genes as computed by the BLAST algorithm are analyzed [41]. In comparison, ANI values are computed based on an all-against-all BLAST comparison of 1020-bp pieces of the nucleotide sequences of the selected genomes as described by Goris et al. (2007) [42]. An ANI or AAI of 97% for a pairwise genome comparison is the currently recommended replacement for the 70% DDH values for species delineation of fungi [43]. The average nucleotide and amino acid identity between two genomes can be used for fungal species delineation, but it is not suitable for genus demarcation. We used the Percentage of Conserved Proteins (POCP) between two strains to estimate their evolutionary and phenotypic distance. A comprehensive genomic survey indicated that the POCP could serve as a robust genomic index for establishing the genus boundary [19,38].

## 3. Results and Discussion

### 3.1. Genomic Data

Within this work, 17 different Ustilaginaceae strains from 14 species were chosen for genome sequencing by application of third-generation Oxford Nanopore sequencing in combination with second-generation Illumina sequencing. Long-read nanopore sequencing was combined with the short-read Illumina sequencing method to improve base accuracy and thus significantly reduce error rates in the final genomes. The strain selection was based on previous work, including relevant species such as *U. cynodontis* [44], *U. trichophora* [45], *U. vetiveriae* [11], and *U. maydis* [6,46]. Several *U. maydis* isolates were included as previous studies determined differences in the secondary metabolite profiles. Apart from the named *Ustilago* species, a broad species selection was aimed for, including *Macalspinomyces* [47], *Pseudozyma* [48], *Sporisorium* [47], and *Ustanciosporium*.

The amount of obtained reads by Nanopore sequencing ranged from 175,216 to 621,889 with an average read count of 350,192, whereas the mean read length shows an average of 13,253 bp (8214–17,321 bp). Assemblies polished by Illumina short reads resulted in an average contig number of 66 (22–316) with a 72-fold average coverage (45.8–95.3 fold). Established genome sequences range in size from 18 to 36 Mb and feature GC contents around 54%, which is common for eukaryotes as they barely reach GC values above 60%. Table 2 shows the assembly results in more detail, incorporating genome size, contig numbers, GC-content, and the number of annotated genes.

A previous study presented draft genome sequences from nine different Ustilaginaceae that are known for itaconic acid production [17]. Nevertheless, so far, no study has focused on a broader spectrum of Ustilaginaceae isolates for whole-genome sequencing, including genomic and phylogenomic analyses.

In summary, the combinatorial approach of Illumina and nanopore sequencing resulted in 17 high-quality and state-of-the-art fungal draft genome sequences that can be used for further analysis.

### 3.2. Phylogenomic Analysis

**Figure 1:**
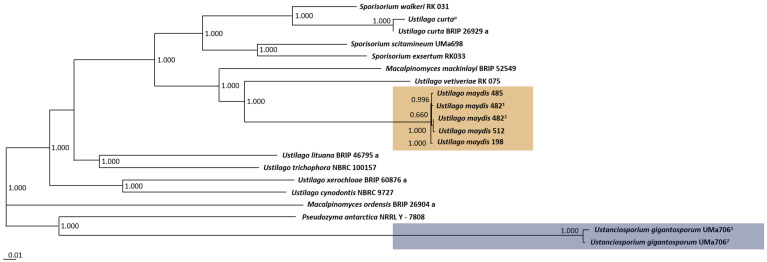
Phylogenetic tree of the investigated Ustilaginaceae strains. The phylogenetic tree is based on the core genes (2370 genes) of the selected strains. The SH-like local support values were computed using FASTtree within the comparative genomics tool EDGAR 3.0 [49]. ^1,2^ Two isolates were sequenced. ^a^ Additional strain isolated from sample #2836* (Table 1).

To deduce the phylogeny of the Ustilaginaceae isolates, the comparative genomics platform EDGAR 3.0 was applied. Based on all core genes determined for the 14 species—in total 2370 genes—a phylogenetic tree was computed (Figure 1). In the case of two strains—*U. maydis* No. 482 and *U. gigantosporum* Uma 706—two isolates were sequenced. Thus, they were labelled with -1 and -2, respectively. The tree consists of six different clusters. In total, two outgroups are visible representing *Pseudozyma antartica* NRRL Y -7808 and *U. gigantosporum* UMa706 (group 1, highlighted in blue), as well as *M. ordensis* BRIP 26904 a (group 2), followed by two clusters consisting of two different *Ustilago* species. The largest cluster consists of all *U. maydis* isolates (highlighted in orange), *U. vetiveriae,* and *M. mackinlayi*. The last cluster includes all *Sporisorium* isolates and *U. curta*.

Previous comparative studies on genomes of smut fungi have indicated that *U. maydis* is more closely related to other taxa than to species of *Ustilago* [1]. Other systematic studies confirmed this fact and further showed that *U. maydis* is closely related to species of *Sporisorium* and *Anthracocystis* [50,51]. The close relationship can be explained by similarities in their host-plant infection mechanism. McTaggart et al. (2016) recovered *U. maydis* in a clade, among others, with *M. mackinlayi*, which all form hypertrophied sori in inflorescences of their hosts [1]. Furthermore, the authors considered that localized, host-derived, hypertrophied sori were an apomorphy for this group [1]. During our phylogenomic analysis based on the core genes of the selected strains, the close relationship between *U. maydis*, *M. mackinlayi*, and *Sporisorium* species was also observed, confirming previous studies (Figure 1). The question remains if *U. maydis* or the other *Ustilago* species require a reclassification.

### 3.3. Comparative Genomics

**Figure 2:**
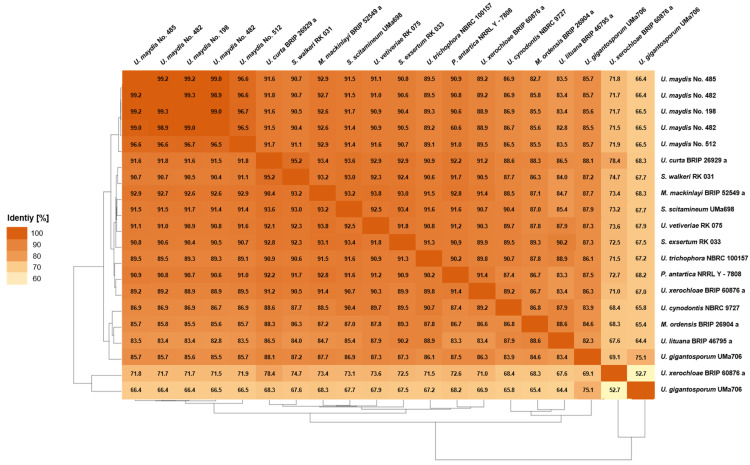
Pairwise Percentage of Conserved Proteins (POCP) analysis for members of the Ustilaginaceae family. The conserved proteins between a pair of genomes were determined by aligning all the protein sequences of one genome (query genome) with all the protein sequences of another genome using the BLASTP program [38]. Proteins from the query genome were considered conserved when they had a BLAST match with an E-value of less than 1 × 10^−5^, a sequence identity of more than 40%, and an alignable region of the query protein sequence of more than 50% [38]. Two isolates were sequenced in case of *U. maydis* No. 482 and *U. gigantosporum* UMa706.

**Figure 3:**
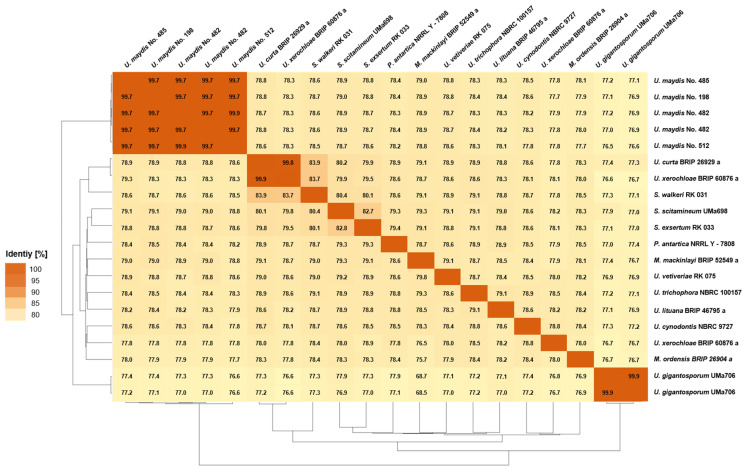
Pairwise Average Nucleotide Identity (ANI) analysis between genome-sequenced Ustilaginaceae. The ANI between the query genome and the reference genome was calculated as the mean identity of all BLASTN matches [42]. Two isolates were sequenced in the case of *U. maydis* No. 482 and *U. gigantosporum* Uma706.

**Figure 4:**
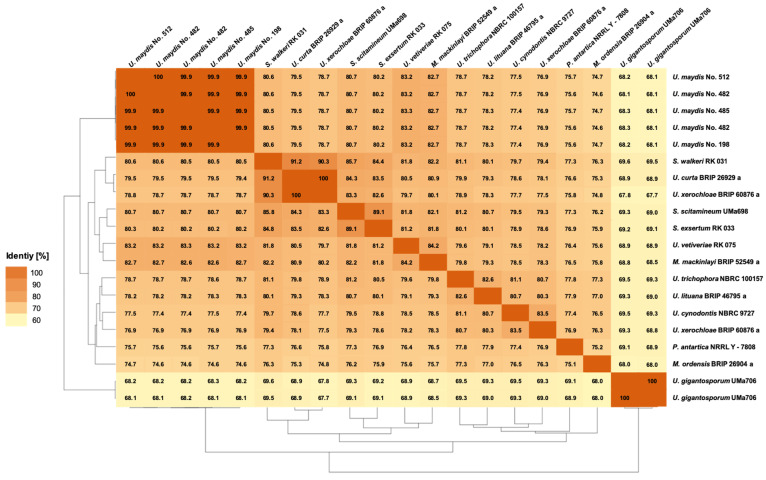
Pairwise Average Amino Acid Identity (AAI) analysis between genome-sequenced Ustilaginaceae. The AAI between the query genome and the reference genome was calculated as the mean identity of all BLASTN matches [42]. Two isolates were sequenced in the case of *U. maydis* No. 482 and *U. gigantosporum* Uma706.

In order to determine the similarities within the Ustilaginaceae species, comparative genomic tools such as Percentage of Conserved Proteins (POCP, Figure 2), Average Nucleotide Identity (ANI, Figure 3), and Average Amino Acid Identity (AAI, Figure 4) were applied to the whole genome sequences.

#### 3.3.1. Pairwise Percentage of Conserved Proteins

The POCP analysis has been proposed as a novel method to define genus boundaries in prokaryotes [38]. In prokaryotic studies, POCP analysis revealed that all pairwise comparisons of species from different families resulted in values lower than 50%, the proposed threshold for a genus boundary. A recent study by Wibberg et al. (2021) proposed that this threshold cannot be directly applied to fungal genomes [19]. Since fungal genes are much more conserved than prokaryotic genes, the threshold was proposed to be set at 70% separating family and non-family members [19]. POCP analysis can be used to compare two strains to estimate their evolutionary and phenotypic distance. Thereby, the quality of POCP analysis depends on the reliability of the applied gene prediction models. Wibberg et al. (2021) showed that protein sequences derived from GeneMark delivered an adequate coverage of the gene content, which is comparable to RNA-Seq-based prediction pipelines [19].

The POCP identities determined in this study ranged from 52–99% (Figure 2). Thereby, the observed identities of <70% could be explained by the impurity of another fungus in samples of *U. gigantosporium* and *U. xerochloae*. Excluding this artifact, the observed identities are comparable to the previously published ones showing POCP identities from 70–99% between the tested family members [19]. The different *U. maydis* strains within one species even share 96–99% identity. The remaining species show identities up to 95%. Nevertheless, *U. maydis* displays the only species that contained several strains within the obtained analysis. In order to verify a potential species threshold, various additional isolates should be genome-sequenced. Differentiation of interspecific and intergeneric identities between the different Ustilaginaceae isolates was not possible, which was also observed in previous studies due to overlapping identities [19]. Thus, our results combined with the phylogenomic analysis (Section 3.2) indicate that a reclassification of the Ustilaginaceae family might be required.

#### 3.3.2. Average Nucleotide Identity

Apart from POCP, ANI (Figure 3) was performed to determine the nucleotide-level genomic similarity between two genomes. The obtained ANI values range from 76.6 to 99.9% identity. Again, the highest identities and similarities were observed for the *U. maydis* isolates ranging from 96.7 to 99.9%. Further, the other comparisons showed a maximum identity of 99.8% for *U. curta* and *U. xerochloae*. Thus, a clear boundary between inter- and intraspecies comparison is not feasible for our obtained data set using ANI analysis only. Furthermore, differentiation of interspecific and intergeneric identities was impossible due to overlapping identities similar to POCP. Wibberg et al. (2021) observed that members of the Hypoxylaceae share at least 70% of their nucleotide content [19]. The different Ustilaginaceae isolates compared during this study showed at least 77% nucleotide content, which is comparable to the previous data received from other fungal families. This threshold is identical to the one estimated for the POCP analysis.

For prokaryotic genome data sets, ANI analyses have been widely used to identify genomic variations and, thus, define species boundaries [52]. Concerning fungal data sets, studies are available for comparably small taxon selections [53]. For selected *Rhizoctonia solani* isolates, an approximate sequence identity of 80% was shown. In a recent study of a larger data set on Hypoxylaceae, a sequence identity within the family of 73.2–93.3% was demonstrated [19]. Moreover, in a study on Arthrodermataceae-related species, identities of 76.4–90.0% were observed, which are comparable to the other studies and our obtained identities (76.6 to 99.9%) [19,54]. When comparing the ANI and POCP between species pairs in the family of Ustilaginaceae, it can be seen that POCP values are overall higher. Comparing *U. curta* BRIP 2629a with *U*. *maydis* isolates, POCP identities of 91.5–91.8 were obtained. ANIs for the comparison resulted in values of 79.5%. A similar phenomenon, higher conservation of protein sequence, was observed for the family of Hypoxylaceae, confirming the obtained results during this study [19].

#### 3.3.3. Average Amino Acid Identity

AAI (Figure 4) measures the genomic difference of the investigated strains based on orthologous proteins [19]. The AAI identities of the different Ustilaginaceae range from 67.7–99.9%, indicating that the strains share at least two-thirds of the presented protein-coding genes. Excluding *U. gigantosporium* and *U. xerochloae* with a contaminated or diploid genome, the identities increase to 74.6–99.9%. The different sequenced *U. maydis* isolates show AAIs ranging from 99–100%. Comparing isolates from different genera, AAIs up to 91.2% were observed, e.g., comparing *U. curta* BRIP 26929 and *S. walkeri* RK031. Comparing the different *U. maydis* isolates and *S. walkeri* RK031 results in AAIs of 80%. *U. xerochloae* BRIP 2629a and *U. maydis* isolates showed identities of 78%. During a previous study, Wibberg et al. (2021) proposed an intergeneric and interfamilial threshold value of 75% for the tested Hypoxylaceae [19]. This study focused on one fungal Ustilaginaceae family only. Nevertheless, AAIs above 74.6% were observed, supporting the findings of the previous study.

In comparison to ANI (Figure 3), the AAIs were higher, which can be explained by the degenerated genetic code as nucleotide sequence changes do not necessarily result in an amino acid change [55].

### 3.4. Gene-Based Comparison

**Figure 5:**
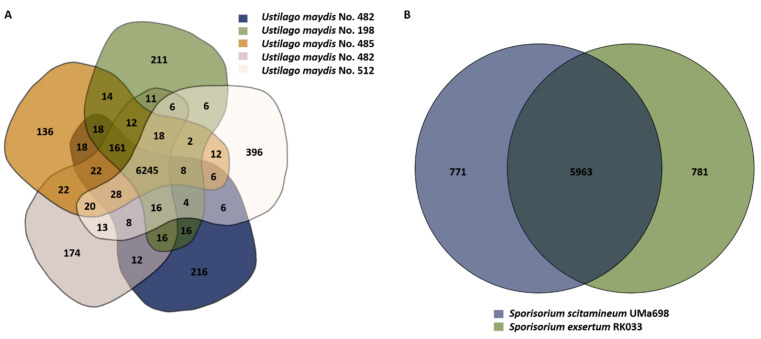
Gene-based comparison of Ustilaginaceae. Venn diagrams of (**A**): *U. maydis* and (**B**): *Sporisorium* isolates obtained from the comparative genomics tool EDGAR 3.0 [49]. Two isolates were sequenced in the case of *U. maydis* No. 482.

Results of the genomic comparison by analyzing the number of core and individual genes between different *U. maydis* and *Sporisorium* isolates, respectively, are displayed in Figure 5. Similar to the POCP, the numbers strongly depend on the accuracy of gene prediction and hence cannot be considered as exact measurements. Nevertheless, it gives insights into the distribution of orthologous genes between related and unrelated species.

*U. maydis* isolates (Figure 5A) share 6245 core genes and contain between 136 (*U. maydis* No. 385) to 396 singleton genes (*U. maydis* No. 512). The number of common genes between the individual pairs varied in the range of 6305 (*U. maydis* No. 512 and 198) to 6578 (*U. maydis* No. 485 and 482).

In addition to the core genome, the regions involved in secondary metabolite synthesis for the different Ustilaginaceae isolates were analyzed and compared (Table 3). In general, these fungi include—also in comparison with other Basidiomycota, e.g., *R. solani* [19]—only a few secondary metabolite synthesis genes and gene clusters, which agrees with previous reports [56]. The number ranges between 10 and 15 regions per genome. Here, the differences between species are not very pronounced. Most of the predicted regions belong to the core genome of the isolates. However, also unique regions were predicted, e.g., the RIPP region in *U. gigantosporum* Uma706 or a second T1PKs region in *M. mackinlayi* BRIP 52549a. Here, additional experiments are needed, such as RNA sequencing experiments to predict the function of the metabolites and to predict if it is involved in the pathogenic interaction with the host plant. Differences between the two respective isolates of *U. maydis* No. 482 and *U. gigantosporum* Uma706 can be explained by separate cultivation and isolation experiment. In the case of eukaryotes, such genetic differences are a common phenomenon. In general, the presented results solely display a prediction on the chosen enzyme families and do not cover all potential secondary metabolites. In the case of polyketide synthases (PKSs), antiSMASH predicts one type 1 PKS, whereas it is known that *U. maydis* has three polyketide synthases (pks3, pks4, and pks5) for the production of the pigment melanin [57]. Indeed, antiSMASH updates are announced to further improve the predictions of secondary metabolite synthesis gene clusters in varying organisms [58]. Another example of missed secondary metabolites are isoprenoids derived from carotenoids—for example, the plant hormone abscisic acid, of which its synthesis was reported for *U. maydis* [59]. The largest class of genes encoding enzymes involved in secondary metabolite synthesis identified here are NRPSs (Table 3). A prominent example of a Ustilaginaceae product requiring NRPS enzymes is the metal chelator ferrichrome [60].

### 3.5. Itaconate Cluster Identification

**Figure 6:**
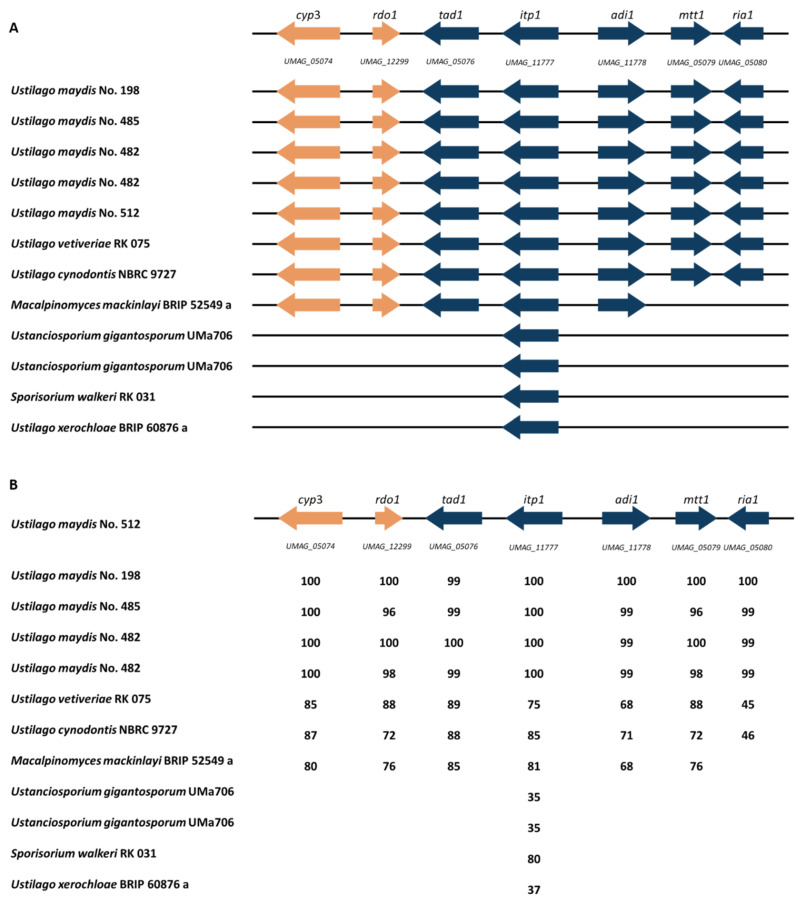
Schematic overview of the itaconate biosynthesis gene cluster in *U. maydis*. Modified from [65] (**A**): Organization of the cluster genes is compared to the sequenced Ustilaginaceae isolates. Blue color indicates genes directly involved in itaconate production and transport [13]. Orange-colored genes are involved in itaconic acid conversion into its derivates [14]. *Cyp3* (P450 monooxygenase), *rdo1* (glyoxalase domain-containing protein RDO1), *tad1* (trans-aconitate decarboxylase 1) *itp1* (itaconate transport protein), *adi1* (aconitate-delta-isomerase 1), *mtt1* (mitochondrial tricarboxylate transporter 1), *ria1* (regulator of itaconic acid biosynthesis). (**B**): Itaconate cluster comparison of selected Ustilaginaceae. Numbers given show amino acid sequence identity as percentage compared to the reference strain *U. maydis* No. 512 using antiSMASH 6.0 [58]. Two isolates were sequenced in the case of *U. maydis* No. 482 and *U. gigantosporum* UMa706.

Based on the obtained genomes, secondary metabolite clusters were investigated in order to verify genetic similarities and differences between the Ustilaginaceae. During previous empirical studies, differences in itaconic acid production were observed for various Ustilaginaceae strains [5]. Thus, the itaconate gene cluster was compared at first (Figure 6). The complete itaconate cluster was identified in six isolates: *U. maydis* No. 198, 485, 482, and 512, as well as in *U. vetiveriae* RK075 and *U. cynodontis*. *M. mackinlayi* BRIP 52549a did not contain *mtt1* and *ria1*, which encode a mitochondrial tricarboxylate transporter and the regulator of itaconic acid biosynthesis, respectively [14]. Thus, itaconate production was tested during a cultivation experiment (Supplementary Figure S1). An itaconate production of 2.2 ± 0.01 g L^−1^ after 48h was observed, indicating a functional itaconic acid synthesis cluster that is also reacting to nitrogen starvation as previously reported for the other *Ustilago* itaconate producer [9]. Previous studies focused on the role of different itaconate cluster genes as, e.g., a deletion of *mtt1* in *U. maydis* MB215 (No. 512) led to a strong decrease in itaconate production, but its production was not completely abolished [14]. This transporter is known as the rate-limiting step in itaconate biosynthesis in *U. maydis* [61]. Most likely in *M. mackinlayi* BRIP 52549a, different less specialized, and therefore less efficient, transport proteins substitute the dedicated itaconic acid transporter, as most eukaryotic mitochondrial transporters have a diverse substrate spectrum with different affinities [62].

Four isolates, *U. gigantosporum* UMa706, *S. walkeri* RK031, and *U. xerochloae* BRIP 60876a, only harbor *itp1*, encoding the itaconate transport protein lacking the remaining itaconic acid cluster genes. This transporter secretes itaconic acid, itatartarate, and 2-hydroxyparaconate from the cytosol to the exterior of the cell [62]. As itaconate reduction was observed in some long-lasting experiments (Supplementary Figure S2), itaconate import might be facilitated by *Itp1*. Indeed, the degradation of itaconate might be important in some niches due to the antimicrobial properties of itaconic acid [63,64]. Geiser et al. [17] presented draft genome sequences of itaconate-producing Ustilaginaceae, including *S. iseilematis-ciliati* BRIP 60887a, *P. tsukubaensis* NBRC 1940, *P. hubeiensis* NBRC 105055, as well as *U. maydis* and *U. vetiveriae* strains. All strains showed the complete itaconate cluster except for *P. tsukubaensis* NBRC 1940. Available genome sequences of investigated Ustilaginaceae strains could be included in the obtained data, which helps facilitate further research on the biology of Ustilaginaceae and increase the list of tools for metabolic engineering of itaconate production by Ustilaginaceae.

To further investigate the itaconic acid cluster genes, amino acid sequence identities were compared (Figure 6B). Each enzyme shares amino acid sequence identities with the corresponding enzymes in *U. maydis* No. 512, with identities ranging from 35–100%. Among those, the highest identities were found for the different *U. maydis* isolates ranging from 96–100%. The lowest identity of 96% is obtained comparing *U. maydis* No. 485 and No. 512 for genes *rdo1* and *mtt1*. *Mtt1* is directly involved in itaconic acid biosynthesis as it encodes a mitochondrial tricarboxylate transporter. *Rdo1* is not directly involved in the itaconic acid synthesis, but it is proposed to encode an enzyme converting (S)-2-hydroxyparaconate to itatartarate [62]. An empirical study by Geiser et al. (2014) confirmed a lower itaconate production of *U. maydis* 485, which might result from genetic differences within the itaconic acid gene cluster [5]. Comparing the different genes, *cyp3* encoding a cytochrome P450 monooxygenase and *itp1* encoding an itaconate transport protein are highly conserved, showing amino acid identities of 100% between the tested *U. maydis* isolates.

For *U. cynodontis* and *U. vetiveriae*, the sequence identity of proteins encoded by the itaconate biosynthesis genes is mostly conserved in a range of 68–89% compared to the *U. maydis* No. 512 sequence. The most divergent protein of the itaconate cluster is Ria1, showing identities from 45–46% for *U. vetiveriae* and *U. cynodontis*, respectively. Geiser et al. observed a similar identity of 43% between *U. maydis* and *U. vetiveriae* even though they are phylogenetically closely related [13]. Further, Geiser et al. (2018) were able to activate silent itaconate clusters by overexpression of Ria1 originating from related species, although the amino acid sequences of Ria1 regulators are less conserved than the enzymes involved in itaconic acid synthesis [13]. Additionally, the authors could enhance itaconate production in weak producers up to 4-fold [13]. Thus, it was shown that activation of silent secondary metabolite clusters could be achieved in a range of related species with reduced genetic engineering efforts.

To conclude, this study confirmed a highly conserved itaconate cluster of the investigated *U. maydis* strains (96–100% identity), even though there are differences in itaconate production and by-product formation such as (S)-2-hydroxyparaconate, which were observed during previous empirical studies. Thus, the obtained results can be used to further increase the list of tools for metabolic engineering of itaconate production by Ustilaginaceae.

### 3.6. MEL Cluster Identification

**Figure 7:**
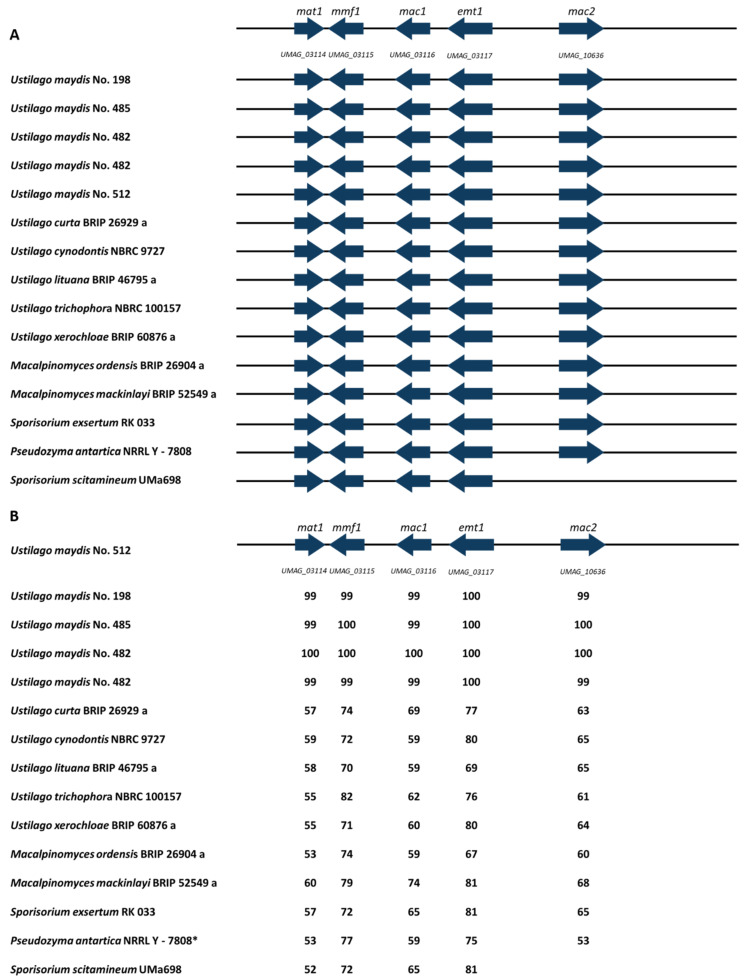
Schematic organization of the MEL synthesis gene cluster (modified from [65]) (**A**): Organization of the gene cluster is compared to the sequenced Ustilaginaceae. *Emt1* (erythritol/mannose transferase), *mac1* and *mac2* (acyl transferases), *mat1* (acetyl transferase), *mmf1* (putative transporter). (**B**): MEL gene cluster comparison of selected Ustilaginaceae. Numbers given represent amino acid sequence identity as percentage compared to the reference strain *U. maydis* No. 512 using antiSMASH 6.0 [58]. Two isolates were sequenced in case of *U. maydis* No. 482.

Mannosylerythritol lipids (MELs) are glycolipid biosurfactants produced by various basidiomycetous yeasts belonging to the genera *Ustilago*, *Pseudozyma*, *Moesziomyces*, and *Sporisorium* [66]. Apart from their interfacial activity, MELs are known to repair damaged human skin and hair. Thus, they have been commercialized in the cosmetics industry for more than a decade [66]. Hewald et al. (2006) [67,68] identified the gene cluster involved in MEL biosynthesis and outlined the pathway for MEL biosynthesis in *U. maydis*. The cluster consists of four enzymes and a transporter: erythritol/mannose transferase (*emt1*), two acyltransferases (*mac1* and *mac2*), acetyltransferase (*mat1*), and the putative transporter (*mmf1*) (Figure 7).

The identification of gene clusters via whole-genome sequencing is essential for the modification of the MEL biosynthesis pathway. Recently, the gene clusters involved in MEL biosynthesis in various *Pseudozyma* strains have been identified by genomic sequencing [6]. The cluster genes were also identified in Ustilaginaceae strains, including *S. graminicola* and *U. hordei*, during recent work [69]. Within this study, 14 isolates exhibited the whole MEL cluster. *S. scitamenium* exhibited all genes except *mac2*, encoding an acyl transferase. Based on the identification of the MEL cluster, new potential MEL producers were reported within this work: *U. curta*, *U. lituana*, *U. trichophora*, *U. xerochloae*, *M. ordensis*, and *M. mackinlayi*. Nevertheless, production levels could differ due to regulation of the secondary metabolite clusters as observed for itaconate production. MEL production of strains *U. cynodontis* and *S. exsertum* was already observed during cultivation experiments [6,69]. Geiser et al. (2014) identified *M. eriachnes* as a natural MEL producer [5]. No MEL cluster genes were identified in *U. gigantosporum*, *U. vetiveriae*, and *S. walkeri*, asking for further analyses into the function of MELs.

Further, amino acid identities between the different MEL cluster genes were compared between *U. maydis* No. 512 and the different Ustilaginaceae strains (Figure 7B). Amino acid identities ranging from 52–100% compared to *U. maydis* No. 512 were computed. A study from Saika et al. (2018) reported similar identities of 54–82% comparing different *Pseudozyma* strains to *U. maydis* No. 512 [6]. The data set obtained during this study extends the existing set with several *U. maydis* strains enabling the interspecies comparison of the different isolates. MEL cluster genes are highly conserved, showing identities ranging from 99–100% for the different *U. maydis* strains. Comparing other *Ustilago* species (such as *U. curta*, *U. cynodontis*, *U. xerochloae*, *U. lituana* and *U. trichophora*) to *U. maydis* No. 512 identities ranging from 55–80% were observed. Comparing the different analyzed *Pseudozyma* strains by Saika et al. (2018), identities from 55–82% were obtained, confirming our intraspecies (intergeneric) observations [6]. Nevertheless, no clear amino acid identity boundary can be set comparing different genera such as *Macalpinomyces*, *Pseudozyma,* and *Sporisorium*.

The obtained genomic and gene cluster data can be used for modifications in MEL production, e.g., via metabolic engineering. Previous studies identified *mac1* and *mac2* as essential genes encoding two acyltransferases for MEL production in *U. maydis* as the deletion strains Δ*mac1* and Δ*mac2* lacked any MEL synthesis [6]. Furthermore, gained knowledge about MEL cluster genes can be used to tailor MEL production in Ustilaginaceae, exploiting more strains than the existing model organisms.

During this study, 14 out of 17 isolates, i.e., 82%, exhibited genes responsible for MEL production. In general, MEL improves the accessibility of hydrophobic nutrients and enhances attachment to nonpolar surfaces. In addition to these more general functions, some biosurfactants also bind heavy metals, display antimicrobial activity, or play an important role in pathogenic development or biofilm formation [70]. The findings in our study display a valuable genomic data set facilitating further understanding of MEL production in different Ustilaginaceae strains.

### 3.7. Ustilagic Acid Cluster Identification

The phytopathogenic basidiomycetous fungus *U. maydis* secretes, under conditions of nitrogen starvation, large amounts of the biosurfactant ustilagic acid (UA). Further, UA displays antibiotic activity [71].

**Figure 8:**
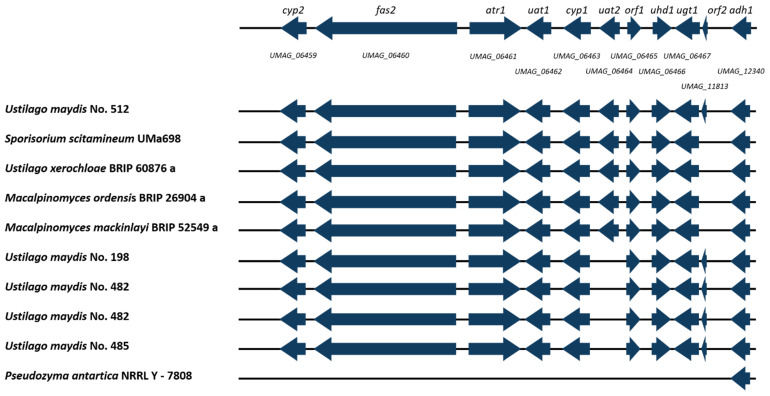
Schematic organization of the ustilagic acid (UA) synthesis gene cluster in *U. maydis* (modified from [65]). Organization of the gene cluster is compared to the here sequenced Ustilaginaceae. *Cyp2* (P450 monooxygenase), *fas2* (fatty acid synthase 2), *atr1* (ABC-type transporter), *uat1* (acyltransferase), *cyp1* (P450 monooxygenase), *uat2* (acetyltransferase), *orf1* (alcohol acetyltransferase), *uhd1* (fatty acid hydroxylase), *ugt1* (glycosyltransferase), *orf2* (ustilagic acid biosynthesis cluster protein), *adh1* (alcohol dehydrogenase I). Gene cluster analysis via antiSMASH 6.0 [58]. Two isolates were sequenced in case of *U. maydis* No. 482.

The genes responsible for UA in *U. maydis* No. 512 production are displayed in Figure 8, which also includes the gene cluster comparison to the other isolates. Based on the given data set, only the reference genome of *U. maydis* No. 512 shows all genes responsible for ustilagic acid production. The remaining *U. maydis* isolates *U. maydis* No. 482, 198, and 485 lack *uat2* encoding an acetyltransferase. Furthermore, another group was identified consisting of *U. xerochloae*, *M. ordensis*, *M. mackinlayi*, *and S. scitamineum* showing all UA cluster genes except *orf2*, which encodes an ustilagic acid biosynthesis cluster protein with a so far unknown function [65]. To conclude, 9 out of 17 isolates (52%) show UA cluster genes. Thereby, this study shows similar findings compared to Geiser et al., confirming *U. maydis*, *S. scitamenieum*, *S walkeri*, and *M. eriachnes* as UA-producing strains during cultivation experiments [5]. Furthermore, smut fungi such as *U. cynodontis*, *U. vetiveriae*, and *S. exertum* did not show UA production during the experiments [5]. Our genomic comparison revealed that this could be explained by the complete lack of the UA cluster. *U. xerocholoae* was identified as a potential novel UA producer as its UA production was not observed during previous studies.

### 3.8. Macro Synteny Plot of U. maydis Isolates

**Figure 9:**
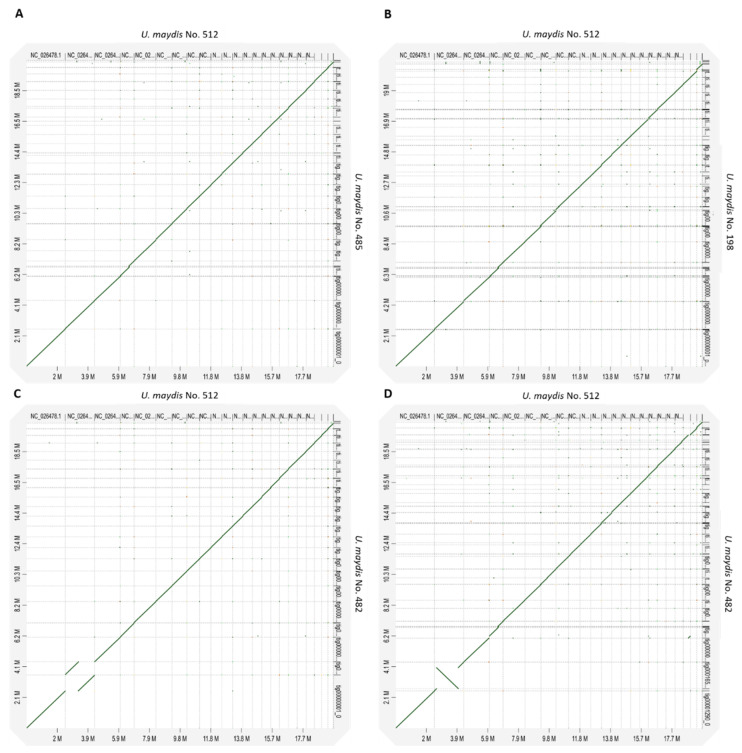
Whole genome macro synteny plot between the different *U. maydis* isolates compared to *U. maydis* No. 512. (**A**) *U. maydis* No. 485 and (**B**) *U. maydis* No. 198. (**C**,**D**) Two isolates were sequenced in case of *U. maydis* No. 482 and therefore are visualized separately.

Genomic synteny of the tested *U. maydis* isolates was compared to *U. maydis* No. 512, the reference strain in biotechnology (Figure 9). When comparing the different isolates, a high genomic synteny can be observed between the respective organism pairs. The chromosomes structure of the *U. maydis* strains is highly conserved. As stated previously, in the case of two strains—*U. maydis* No. 482 and *U. gigantosporum* Uma 706—two isolates were sequenced. Thus, they were labeled with -1 and -2, respectively. Only the two isolates of *U. maydis* No. 482 show different rearrangements of the second chromosome. Differences between the two isolates might result from separate cultivation, assembly errors, or different read length in the respective region. Nevertheless, the two isolates show rearrangements in the same region of the second chromosome which indicates a difference to chromosomes of *U. maydis* No. 512.

To conclude, the highly conserved chromosome structures of the *U. maydis* strains confirm the high identities obtained from POCP, AAI, and ANI resulted in high identities between the different strains.

## 4. Conclusions

Here, we reported high-quality genomes of 17 Ustilaginaceae strains covering 14 species combining nanopore and Illumina sequencing. A broad strain selection for genomic investigation was aimed for based on previous work on relevant Ustilaginaceae species (i.e., *U. cynodontis* [44], *U. trichophora* [45], *U. vetiveriae* [11], *U. maydis* [6,46]). By phylogenomic comparison and the application of taxonomical genome-based bioinformatics methods such as POCP, ANI, and AAI analyses on a larger set of related fungal species, we gained insights into their relationships and were able to deduce taxonomic hierarchies. The obtained results indicate that a reclassification of the Ustilaginaceae family might be required. In addition to genomic comparisons, secondary metabolite cluster analysis was performed. The numbers of identified secondary metabolite clusters did not differ much between species and ranged between 10 and 15 regions per genome. Here, the differences between the different isolates were not very pronounced. Notably, few of the identified regions could be associated with the secondary metabolite synthesized, asking for additional experiments such as RNA sequencing to predict the function of the metabolite and to predict if it is involved in the pathogenic interaction with the host plant.

With the presented high-quality draft genomes (N50 > 1 Mb), the investigation of Ustilaginaceae biodiversity is enabled, as well as metabolic engineering of secondary metabolites production (e.g., MELs, UA, itaconic acid) is fostered. The generated genomic data set enhances our toolbox for investigating and engineering the fungal Ustilaginaceae family.

## Figures and Tables

**Table 1:** DDBJ/EMBL/GenBank database accession numbers.

Strain No.	Assembly Name	Assembly ACC	Study ID	Sample ID	Contig ACC
#2169	Umay_482_v1	GCA_928722285	PRJEB50355	ERS10392836	CAKMXG010000001-CAKMXG010000046
#2169	Umay_482_v2	GCA_928722265	PRJEB50355	ERS10392837	CAKMXF010000001-CAKMXF010000068
#2172	Umay_485	GCA_928722245	PRJEB50356	ERS10381369	CAKMXD010000001-CAKMXD010000043
#2136	Umay_198	GCA_928743665	PRJEB50359	ERS10419843	CAKMXO010000001-CAKMXO010000107
#2816	BRIP_26904_a	GCA_928724745	PRJEB50498	ERS10395116	CAKMXJ010000001-CAKMXJ010000042
#2701	NBRC_100157	GCA_928724775	PRJEB50365	ERS10395119	CAKMXL010000001-CAKMXL010000048
#2212	RK_033	GCA_928724755	PRJEB50360	ERS10395351	CAKMXK010000001-CAKMXK010000041
#2215	UMa706_1	GCA_928724875	PRJEB50363	ERS10395376	CAKMXN010000001-CAKMXN010000086
#2215	UMa706_2	GCA_928724795	PRJEB50363	ERS10395377	CAKMXM010000001-CAKMXM010000036
#2814	BRIP_52549_a	GCA_928852645	PRJEB50497	ERS10422304	CAKMXS010000001-CAKMXS010000079
#2821	BRIP_26929_a	GCA_928722295	PRJEB50499	ERS10393563	CAKMXH010000001-CAKMXH010000039
#2214	RK031	GCA_928858565	PRJEB50362	ERS10422305	CAKMXV010000001-CAKMXV010000022
#1946	NRRL_Y-7808	GCA_928872045	PRJEB50496	ERS10422313	CAKMYB010000001-CAKMYB010000027
#2706	NBRC_9727	GCA_928865425	PRJEB50366	ERS10422385	CAKMXY010000001-CAKMXY010000048
#2826	BRIP_46795_a	GCA_928869825	PRJEB50500	ERS10422474	CAKMYA010000001-CAKMYA010000069
#2836	BRIP_60876_a	GCA_928856705	RJEB50501	ERS10422499	CAKMXT010000001-CAKMXT010000026
#2836 *	-	ERZ4998446	PRJEB50495	ERS10422500	ERZ4998446.1-ERZ4998446.260
#2213	UMa698	GCA_928991175	PRJEB50361	ERS10422257	CAKMYD010000001-CAKMYD010000051
#2220	RK_075	GCA_928722275	PRJEB50364	ERS10392838	CAKMXE010000001-CAKMXE010000037

* additional strain identified in sample #2836.

**Table 2:** Details of the genome sequences of the selected Ustilaginaceae. ^a^ as identified via GeneMark tool, * diploid/ + additional fungi.

No.	Organism	Strain	Genome Size (bp)	Contigs	Largest Contig	N50 (bp)	GC (%)	Annotated Genes ^a^
#2814	*Macalpinomyces mackinlayi*	BRIP 52549a	20,011,713	79	2,517,462	778,176	55.2	6780
#2816	*Macalpinomyces ordensis*	BRIP 26904a	21,488,978	42	1,934,029	921,621	54.4	7166
#1946	*Pseudozyma antarctica*	NRRLY 7808	18,256,718	27	2,397,276	72,913	60.8	6532
#2212	*Sporisorium exsertum*	RK 033	19,675,720	41	1,762,232	1,163,924	56.7	6772
#2213	*Sporisorium scitamineum*	UMa698,	20,280,126	51	1,999,406	881,538	54.9	6757
#2214	*Sporisorium walkeri*	RK 031	18,415,360	22	2,584,725	1,158,577	53.9	6584
#2215	Ustanciosporium gigantosporum *	UMa706	26,778,318	86	3,628,339	1,232,426	55.3	8712
#2215	*Ustanciosporium gigantosporum*	UMa706	20,177,783	36	3,087,333	1,238,668	52.0	6690
#2821	*Ustilago curta*	BRIP 26929a	18,446,555	39	1,762,630	682,062	55.2	6420
#2706	*Ustilago cynodontis*	NBRC 9727	23,130,474	48	2,786,107	1,026,819	52.1	7322
#2826	*Ustilago lituana*	BRIP 46795a	25,770,532	69	1,993,860	819,494	54.3	7741
#2136	*Ustilago maydis*	No. 198	21,122,121	107	2,522,778	660,941	53.8	6807
#2169	*Ustilago maydis*	No. 482	20,585,367	46	3,568,257	87,601	54.0	6827
#2169	*Ustilago maydis*	No. 482	20,591,394	68	2,499,251	674,905	53.9	6833
#2172	*Ustilago maydis*	No. 485	20,582,581	43	2,514,809	743,668	53.9	6777
#2701	*Ustilago trichophora*	NBRC 100157	20,713,809	48	1,974,389	794,480	53.8	6585
#2220	*Ustilago vetiveriae*	RK 075	18,373,116	37	2,393,662	780,848	54.8	6347
#2836	Ustilago xerochloae *	BRIP 60876a	36,081,614	315	4,586,684	648,688	54.8	11,418

**Table 3:** Investigation of secondary metabolite synthesis via antiSMASH analysis [58]. ^1,2^ Two isolates were sequenced, contains * diploid/+ additional fungi.

Organism	Strain	NRPS	Terpene	T1PKS	RIPP	Total
*Ustilago maydis*	No. 485	11	2	1	0	14
*Ustilago maydis*	No. 482 ^1^	11	2	1	0	14
*Ustilago maydis*	No. 482 ^2^	13	2	1	0	15
*Ustilago vetiveriae*	RK 075	7	2	1	0	10
*Ustilago curta*	BRIP 26929a	7	3	1	0	11
*Macalpinomyces ordensis*	BRIP 26904a	6	3	1	0	10
*Ustilago trichophora*	NBRC 100157	7	2	1	0	10
*Sporisorium exsertum*	RK 033	8	3	1	0	12
*Ustanciosporium gigantosporum*	UMa706 ^1^	7	4	1	1	13
*Sporisorium scitamineum*	UMa698	9	3	1	0	13
*Ustilago xerochloae*	BRIP 60876a *	8	2	1	0	11
*Ustilago xerochloae*	BRIP 60876a *	5	2	0	0	7
*Macalpinomyces mackinlayi*	BRIP 52549a	7	3	2	0	12
*Ustanciosporium gigantosporum*	UMa706 ^2^	7	2	1	0	10
*Sporisorium walkeri*	RK 031	6	3	1	0	10
*Ustilago maydis*	No. 198	11	2	1	0	14
*Ustilago cynodontis*	NBRC 9727	9	2	1	0	12
*Ustilago lituana*	BRIP 46795a	6	3	1	0	10

## Data Availability

The genome data of used fungi have been deposited in the DDBJ/EMBL/GenBank database (Table 1). The version described in this paper is the first version.

## Supplementary Materials

The following are available online at https://www.mdpi.com/article/10.3390/jof8030269/s1, Figure S1: Shake flask cultivation experiment of *M. mackinlayi* BRIP 52549a, Figure S2: Controlled-batch fermentation of *U. maydis* No. 512, Table S1: Ustilaginaceae strains screened in this study.
