# Supplementary material for: Seventeen Ustilaginaceae High-Quality Genome Sequences Allow Phylogenomic Analysis and Provide Insights into Secondary Metabolite Synthesis
Source: J Fungi (Basel). 2022 Mar 8;8(3):269. doi: 10.3390/jof8030269 (PMC8951962; doi:10.3390/jof8030269)
Supplement: Supplementary file 1 [file jof-08-00269-s001.zip › jof-1611494-supplementary.pdf]

Supplementary materials

Supplementary figures

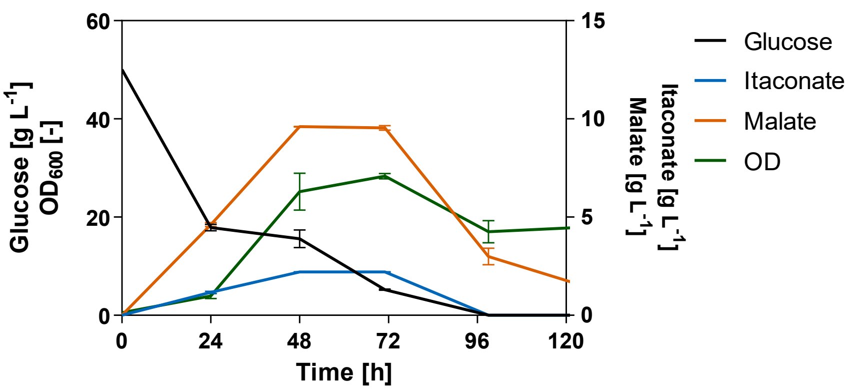

Figure S1 Shake flask cultivation experiment of *M. mackinlayi* BRIP 52549a. Growth via OD_{600} measurement (green line), glucose concentration (black line), malate production (orange line) and itaconate production (blue line) are visualized during small scale (50 ml) cultivation experiment using MTM medium and 50 g L^{-1} glucose and 0.8 g L^{-1} NH_4Cl. Error bars indicate the deviation from the mean for n=3.

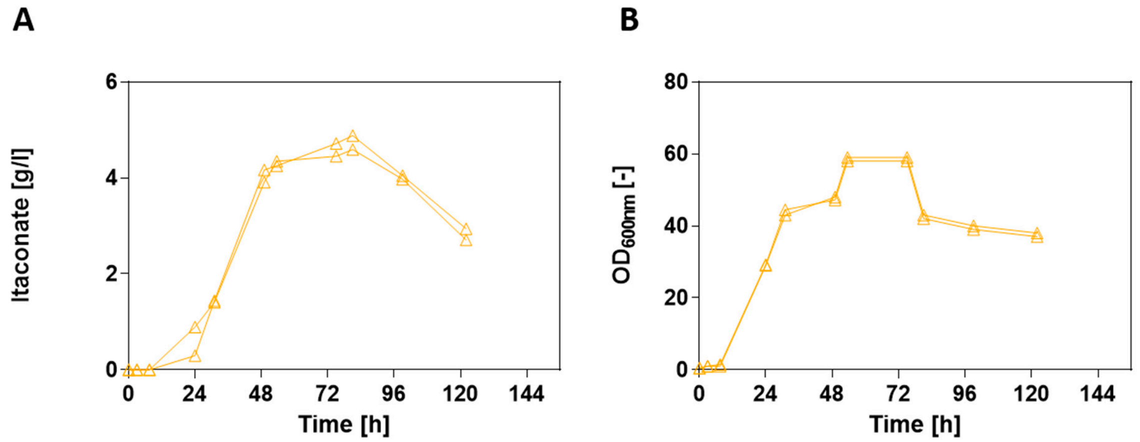

Figure S2 Controlled-batch fermentation of *U. maydis* No. 512. A: Itaconate production and B: growth via OD_{600} measurement on in a bioreactor containing MTM medium using 50 g L^{-1} glucose, 6.25 g L^{-1}, 0.8 g L^{-1} NH4Cl, 80% DOT, at pH 6.5 [1].

Supplementary tables

Table S1 Ustilaginaceae strains screened in this study. Numbers (#) indicate the strain number.

No.	Strain		Origin
#2169	*Ustilago maydis*	Nr. 482	Prof. M. Bölker, Philipps University Marburg, Germany
#2172	*Ustilago maydis*	Nr. 485	
#2136	*Ustilago maydis*	Nr. 198	
#2212	*Sporisorium exsertum*	RK 033	CBS 133249
#2213	*Sporisorium scitamineum*	UMa698, Sscl4, JS109, RK 109, Mating type MAT1	CBS 131457
#2214	*Sporisorium walkeri*	RK 031	CBS 131462
#2215	*Ustanciosporium gigantosporum*	UMa706	CBS 131464
#2220	*Ustilago vetiveriae*	RK 075	CBS 131474
#2701	*Ustilago trichophora*		NBRC 100157
#2706	*Ustilago cynodontis*		NBRC 9727
#2708	*Ustilago rabenhorstiana*		NBRC 8995
#1946	*Pseudozyma antarctica*		NRRL Y - 808
#2814	*Macalpinomyces mackinlayi*	BRIP 52549 a	Queensland Plant Pathology Herbarium, Australia
#2816	*Macalpinomyces ordensis*	BRIP 26904 a	
#2821	*Ustilago curta*	BRIP 26929 a	
#2826	*Ustilago lituana*	BRIP 46795 a	
#2836	*Ustilago xerochloae*	BRIP 60876 a	

Supplementary references

1. Ullmann, L.; Phan, A.N.T.; Kaplan, D.K.P.; Blank, L.M. Ustilaginaceae Biocatalyst for Co-Metabolism of CO2-Derived Substrates toward Carbon-Neutral Itaconate Production. *J. Fungi* **2021**, *7*, 98.
